# Prolapsus Uteri

**Published:** 1855-12

**Authors:** W. Stone


### *Prolapsus Uteri.*

Messrs. Editors: The following extract from a published lecture by Dr. Barker, Professor of Midwifery and Diseases of Women and Children, I think is worthy of the consideration of the profession. If you agree with me in opinion, please insert it in Journal:

"In the beginning of the lecture, following Vidal, I divided the causes of prolapsus into those inherent to the uterus itself, (that is, all those causes which augment the volume or weight of the uterus,) those which refer to the state of the vagina and pelvis, and those which refer to the attachments of the uterus.

"Now, the indications for cure must be based on the causes which produce the displacement. Prolapsus, resulting solely from the condition of the uterus itself, usually, I think, subsides spontaneously when the uterine trouble is removed. I have often found the uterus very low in the pelvic cavity when there is inflammatory disease of the cervix, but rising higher and higher during the treatment. I may add that, so far as my experience goes, this is a much more frequent cause than both the others; but it is to prolapsus depending upon the other causes to which I wish now more especially to call your attention. That there are causes entirely independent of the weight of the uterus, I am perfectly certain; as I have in several instances seen *complete prolapsus* where the uterus was evidently atrophied. Admitting then, both the causes before mentioned, the indications for cure will be, 1st, to retain the uterus in its normal condition; 2d, to diminish the preternatural capacity of the vagina; and 3d, to restore tone to the ligaments. The first is gained by mechanical means, the second by astringents, and the third by fulfilling the first two, and increasing the tgeneral vital powers. Very slight support suffices to fulfil the firts. I have rarely found any evil resulting from the attempt to accomplish the second, such as injury to the general health from arrest of the accustomed discharges, or inflammation or irritation of the mucous membrane of the vagina. The method which I adopt is the following: I cut out a double thickness of patent lint of a triangula form, so that when rolled up it will form a cone, of a size adapted as nearly as I can judge to the capacity of the vagina. Half an inch from the apex is firmly tied a piece of narrow bobbin, for the purpose of facilitating withdrawal. This is soaked in a saturated solution of tannin. The patient being placed upon her back, the uterus is replaced, care being taken to adjust it so that its axis corresponds with the axis of the superior strait, and the lint introduced with the apex first; but after it is in the vagina it is turned, so that the base will come under the os tincæ. This is withdrawn, and a new one introduced, twice in the twenty-four hours. In some cases there is soreness and tenderness of the vagina, when I add to each ounce of the solution of tannin        of laudanum. I have used morphine, but the laudanum seems to be more efficient in removing the soreness. The size of the lint pressary is gradually diminished until the base is not more than half an inch in diameter, when the cure may be considered as accomplished. This should never be left for the patient to do herself. It requires the personal attendance of the physician. The patient will not do it properly or efficiently. You will surely be disappointed if you trust her. If she have means, she will not demur at paying for all the trouble you are at in effecting a cure; if she be poor, you will be amply repaid in seeing her able to perform her duties in life with comfort and ease.

"This is a very different mechanical support from the sponge, which expands in the vagina, or any unyielding pessary, or even the sachet 'filled with finely grained, not pulverized, Aleppo galls,' of which Professor Meigs speaks. This *contracts* in the vagina. It, so to speak, *packs* in the vagina, so that when you withdraw it, you will find it much smaller than when you introduced it. Indeed, I am sure you will be surprised to find how rapidly you are obliged to diminish the size of the lint. But local treatment is not all that is necessary. I need hardly say that, previous to commencing this treatment, the bowels should be thoroughly evacuated, and that during the whole treatment they should be kept *well opened*.—Every man of tact and discrimination will adapt this general treatment to the peculiarities of his patient. Many of this class require tonics. To some I have given three times a day two grains of quinine, in a wine-glass full of the solution of the citrate of magnesia. To others I have given the tart or the citrate of iron in the same solution. Some I have given the iodide of iron, and recently I have been greatly pleased with the effects of the maganese as a tonic. All do not require tonics. But above all things keep the bowels open, and even after you cease attendance, threaten your patient with all the terrors of a relapse, if she do not keep her bowels open.

"Formerly I used to direct my patients to keep the recumbent posture during the first week of treatment; but on finding that my poor patients, who were obliged to keep about, got along better than those in better circumstances, I have now adopted a different course, and send them out into the open air as much as possible, from the beginning.

"This mode of treatment is applicable to each of the different degrees of prolapsus. I have before remarked that the symptoms, where there is but slight depression, are quite as severe in some, as those attending complete prolapsus are in others. I have several times been led to suspect, from the severity of the symptoms complained of, that inflammatory disease of the cervix existed; but a careful examination with the speculum revealed no disease. The pain in the back, nausea, fever, vaginal irritation, and constipation, when the result of the depression. Lisfranc declares that all cases of incipient prolapsus are caused by congestion. He directs that the congestion of the uterus should be first treated, and if after that the displacement of the womb continues, the pessary may be applied if the patient can bear it. Now, the lint and tannin pessary applied in the manner in which I have directed, relieves this condition of things at once. To borrow an illustration from Dr. Meigs, it acts like a suspensory in the treatment of orchitis.

"In complete prolapsus, you will be able to use this means of treatment when no other form of pessary can be retained or worn. Sometimes it will be necessary for the patient to wear for a time a perineal bandage, but this is not often the case. Please to try this method, and see if you can not in all cases effect a radical cure. I believe you can, in all cases excepting those where the sacrum is very straight, and there has been great loss of the substance of the perineum.

"I could give you the history of many cases of complete prolapsus, where a perfect and radical cure has been effected by this plan. A widow, aged thirty-two, cook for a large and fashionable boarding-house in University Place, had the uterus entirely protruded from the vagina. She was obliged to wear a napkin constantly, to keep the uterus within the vulva. It protruded at once on removing the napkin. She was cured in two months, by the plan I have described. Soon after, she married a waiter in the house. The third of July she went on a steamboat excursion, and danced a good deal. I was called to see her on the fourth, on account of a severe flooding; and she miscarried with a five month fœtus. She resumed her duties as cook within a week, but there was no return of the prolapsus.

"An old lady, sixty-eight years of age, residing in Columbia street, had suffered with complete prolapsus for more than twenty years.—Various kinds of pessaries had been at different times adjusted by men of eminence in this city, but for five years she had been unable to wear any. I found her in bed—where she passed the greater part of the time—the uterus small, but the whole tumor external to the vulva, formed by the uterus, vagina, part of the bladder, and part of the rectum, was as large as the egg of a goose. If the tumor was pushed back while lying on her back, it immediately returned. The mucous membrane of the vagina was superficially ulcerated in two places, in one to the size of a twenty-five cent piece, and the other considerably smaller. The cure was effected in three months. The last time I saw her, she said that there was no tendency to falling; and she had left off for some weeks the perineal bandage which I had made for her.

I am often asked, by friends with whom I have conversed in regard to this plan of treatment, if I have never met with evil consequences from the suppression of the profuse discharge which usually attends the prolapsus? I have, two or three times, but not within the last five years. Formerly I was less careful than now, to use laxatives *freely* during the whole course of treatment."

The remarks of the author upon the importance of avoiding constipation should be particularly borne in mind. From many causes the female more frequently suffers from constipation than the male, and the consequences are the more serious as she has an additional and important organ to be influenced by it. Constipation and its consequences to the rectum itself not only produces many of the symptoms that are, in this age of uterine hobbies, referred to the uterus, but is a fruitful cause of prolapsus. Tenesmus and persistant bearing down, whether it is caused by constipation or the state of the uterus itself is the real or most common cause of prolapsus. W. STONE.

[*New Orleans Medical News & Hospital Gazette.*

